# Ethylene Oxide Exposure Attribution and Emissions Quantification Based on Ambient Air Measurements near a Sterilization Facility

**DOI:** 10.3390/ijerph17010042

**Published:** 2019-12-19

**Authors:** Eduardo P. Olaguer, Amy Robinson, Susan Kilmer, James Haywood, Doreen Lehner

**Affiliations:** Michigan Department of Environment, Great Lakes, and Energy, Lansing, MI 48909, USA; robinsona1@michigan.gov (A.R.); kilmers@michigan.gov (S.K.); haywoodj@michigan.gov (J.H.); lehnerd@michigan.gov (D.L.)

**Keywords:** ethylene oxide, ambient measurements, inverse modeling, exposure, risk assessment

## Abstract

Ethylene oxide (EtO) is a known carcinogen and mutagen associated with increased incidence of breast and blood cancers. The largest medical sterilization facility in Michigan had been assessed by the U.S. Environmental Protection Agency as imposing an additional cancer risk greater than one in one thousand in nearby neighborhoods. This prompted the Michigan Department of Environmental Quality (now referred to as the Department of Environment, Great Lakes, and Energy) to conduct an air quality modeling study of the ambient EtO impacts of the sterilization facility, followed by 24 h Summa canister sampling and TO-15 analysis in two phases. Inverse modeling of the measured 24 h EtO concentrations during the second phase yielded estimates of 594 lbs/year for the facility’s total emissions of EtO and 0.247 µg/m^3^ for the urban background concentration. The inverse-modeled emissions are similar to reported emissions by the facility operator based on indoor air measurements and simple mass balance assumptions, while the inferred background concentration agrees with estimates from other field investigations. The estimated peak 24 h exposure to EtO caused by the sterilization facility in nearby neighborhoods was 1.83 μg/m^3^ above the background level, corresponding to an additional cancer risk of approximately one in one hundred, if assumed to represent annual mean exposure.

## 1. Introduction

Ethylene Oxide (EtO), a flammable, colorless gas first described by the French chemist Charles-Adolphe Wurtz, is produced for commercial use mainly by the catalytic oxidation of ethylene. It is often employed in lieu of steam to sterilize delicate, heat-sensitive medical devices that contain plastics or electronics. Ethylene oxide was first applied by the United States military as a sterilant in 1940 and later patented for the sterilization of medical equipment in 1950 [1].

Only about 0.05% (according to 2004 data) of globally produced EtO is used for sterilization or fumigation [1]. About 73% of EtO is used as an intermediate in the manufacture of other products, the bulk of which are ethylene glycols. Other chemicals produced by EtO include ethoxylates, ethanolamines, glycol ethers, polyethylene glycols, and polyethylene polyols. These products are used as polyester fibers, fiberglass, and plastic packaging film. 

In 2009, the world production of EtO was 20 million metric tons [1]. In 2016, United States aggregate production volumes for EtO were estimated at 10 billion lbs (~4.5 million metric tons) [2]. In 2018, EtO was primarily produced in Northeast Asia, the Middle East, and North America. The Middle East increased the production of EtO by 3.4 million metric tons between 2005 and 2010 because of the abundance of ethane in the region [3]. Over 1 million metric tons/year of EtO is manufactured and/or imported in the European Economic Area [4].

Human exposure to EtO is associated with an increased incidence of breast and blood cancers, including multiple myeloma, leukemia, Hodgkins lymphoma, and non-Hodgkins lymphoma [5]. In June 1985, the United States Environmental Protection Agency (USEPA) assessed EtO as a probable human carcinogen and assigned an Inhalation Unit Risk (IUR) to EtO of 1.0 × 10^−4^ per µg/m^3^ based on animal data [6]. The 1990 revision of the U.S. Clean Air Act (CAA) included EtO as a Hazardous Air Pollutant (HAP) in Section 112(d). The National Emission Standards for Hazardous Air Pollutants (NESHAP) established by the CAA subjected EtO to a Maximum Achievable Control Technology (MACT) standard, as well as a subsequent Risk and Technology Review to determine whether additional controls at regulated facilities are necessary to reduce unacceptable residual risk.

In December 2016, the USEPA significantly raised the EtO IUR to 3 × 10^−3^ per µg/m^3^ based on an epidemiological study by Steenland and others [7,8] and concluded that EtO is carcinogenic by a mutagenic mode of action [9,10]. In 2017, the Michigan Department of Environmental Quality (MDEQ), currently known as the Department of Environment, Great Lakes, and Energy (EGLE), updated its Initial Risk Screening level (IRSL) for EtO from an annual average concentration of 0.03 µg/m^3^ to an annual average concentration of 0.0002 µg/m^3^, corresponding to a cancer risk level of approximately one in a million.

In August 2018, the USEPA [11] released the 2014 National Air Toxics Assessment (NATA), which made use of emissions reported by industrial facilities in 2014 to estimate human exposure and risk from inhalation of HAPs. Among the industrial sites that imposed an additional cancer risk greater than one in one thousand in the 2014 NATA was the largest medical sterilization facility in Michigan, located in the city of Grand Rapids. Prior to the release of the 2014 NATA, the facility (which began using EtO as a sterilizer in 1984) had already been issued a Violation Notice by MDEQ for failure to achieve an NESHAP-required 99% reduction in sterilization-chamber and aeration-room vent gas emissions of EtO and a minimum capture and destruction efficiency of 99.5%.

**Figure 1:**
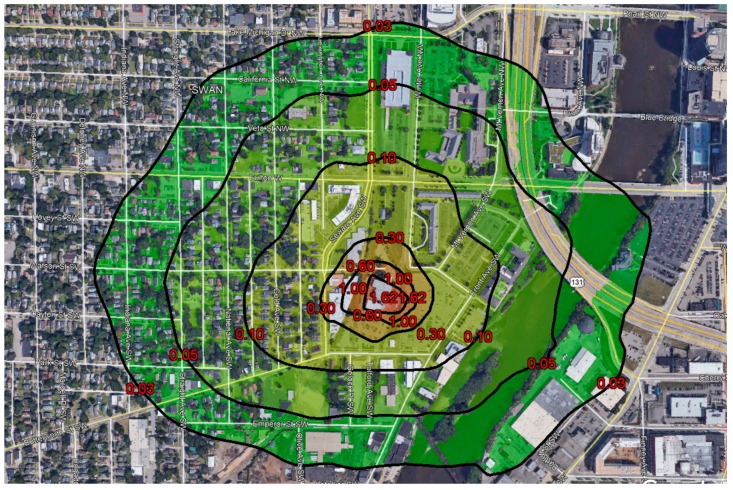
Modeled annual mean ethylene oxide (EtO) concentration (µg/m^3^) resulting from the sterilization facility’s reported emissions for January through August 2018.

In October 2018, MDEQ conducted an air dispersion modeling assessment of the sterilization facility’s reported actual emissions for January through August 2018. Fugitive emissions were estimated by the sterilization facility staff based on gas chromatograph measurements of indoor air and simple mass balance assumptions, while stack emissions were estimated via stack tests using Fourier Transform Infrared Spectroscopy (FTIR). The modeling assessment was performed by MDEQ using the AERMOD v18081 Gaussian plume model [12,13], assuming a constant average hourly emission rate and using five years of hourly meteorological data from a nearby airport. MDEQ [14] inferred a maximum annual average ambient air concentration of EtO in residential neighborhoods around the sterilization facility of ~0.3 µg/m^3^ (see Figure 1), which is 10 times the previous IRSL and 1500 times the current IRSL. This exposure was predominantly due to uncaptured fugitives (i.e., emissions not controlled by a scrubber or subsequently vented through a stack) from the shipping room vent on the east side of the facility.

In subsequent sections of this paper, we describe the results of an ambient air monitoring campaign conducted by MDEQ around the sterilization facility in the immediate aftermath of the 2018 modeling study and an inverse modeling analysis to estimate fugitive emissions from the sterilization process based on the MDEQ ambient air measurements. Our aims are: (1) to evaluate the veracity of reported EtO emissions from the sterilization facility using an independent method and (2) to demonstrate what portion of ambient exposure to EtO in residential neighborhoods can be attributed to the facility as opposed to background sources. As far as we know, this is the first ever attempt to apply inverse modeling to EtO measurements in order to address these issues.

## 2. Ambient Air Monitoring Study

The only widely available method for measuring ambient concentrations of EtO is based on Summa canister sampling and off-line laboratory analysis using USEPA Method TO-15, refined to eliminate interference from trans-2-butene, as described in Ramboll [15]. While it is possible to perform real-time ambient air measurements of EtO using proton-transfer-reaction–mass spectrometry with detection limits down to 100 ppt [16,17,18], the cost of doing this is not normally within the budgets of state and local regulatory agencies.

The MDEQ contracted with Eastern Research Group (ERG) to analyze 24 h EtO Summa canister samples using Method TO-15 with a detection limit of 0.0819 µg/m^3^, which is above the IRSL established by MDEQ, so that non-detects do not necessarily indicate the lack of significant EtO exposure. Canister sampling was conducted in two phases. Phase I took place from ~12:00 Local Standard Time (LST) on November 29, 2018 until ~12:30 LST on November 30, 2018. Phase II was conducted from ~12:00 LST on March 27, 2019 to ~12:00 LST on March 28, 2019. 

The MDEQ staff filled out a Chain of Custody form for each canister sample, as well as a Canister Sampling Field Test Data Sheet. Moreover, each site was photographed for documentation. Once collected, the canisters were brought to a warehouse in Lansing, Michigan, to be prepared for overnight shipping back to the ERG laboratory. The laboratory Chain of Custody forms and the Canister Sampling Field Test Data Sheet were sent to ERG along with the canisters, with separate copies retained by MDEQ.

**Figure 2:**
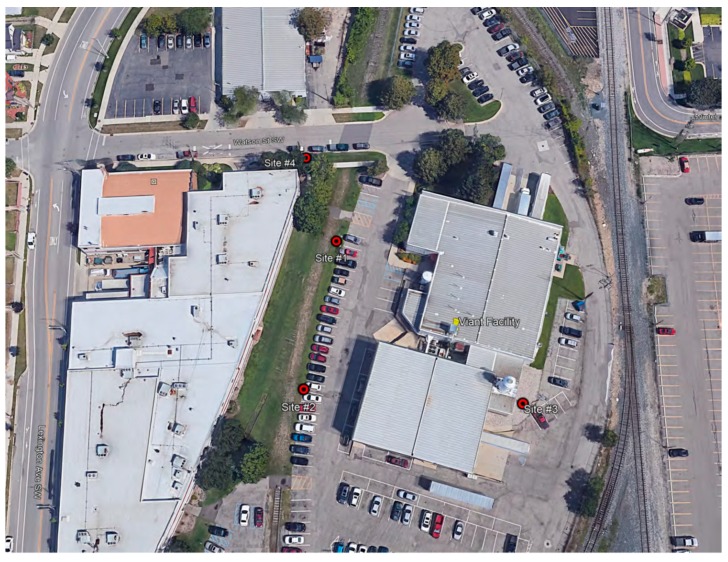
Summa canister sampling sites (red markers) for Phase I.

**Figure 3:**
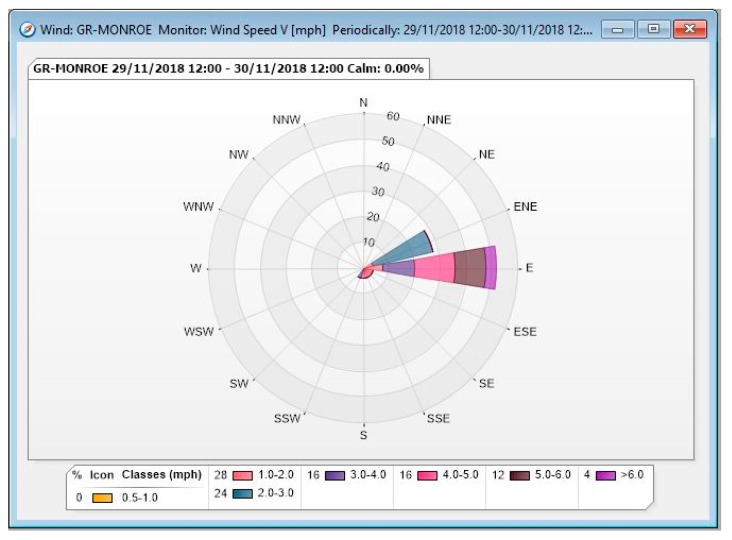
Wind rose for Phase I sampling period based on data from the Michigan Department of Environmental Quality (MDEQ) Grand Rapids—Monroe monitoring station.

During Phase I, five Summa canisters with fixed orifices were set up at four sites around the sterilization facility, including a co-located canister at one site to identify bias in the collection analysis. The four sites are illustrated in Figure 2, while Figure 3 shows a wind rose for the relevant time period. Three of the Phase I sites were on the facility property, while the fourth was at a nearby public sidewalk. After the samples were collected and prepared for shipment, they were sent to the ERG laboratory on December 4, 2018. 

Table 1 summarizes the results of the Phase I campaign. The maximum 24 h average EtO concentration measured during Phase I was 76.0 µg/m^3^. The location of the maximum concentration was just east of the shipping room vent on the sterilization facility’s property (Site #3 in Figure 2), as predicted by the earlier dispersion modeling assessment. The measured 24 h maximum was greater than the model-predicted 24 h maximum of 50.0 µg/m^3^.

The lowest 24 h EtO concentration measured during Phase I was 0.42 µg/m^3^ at a location just outside the northern edge of the facility, in what may be considered ambient air accessible to the general public (Site #4 in Figure 2). The dispersion model predicted a factor of 10 decrease between the 24 h concentration and the annual concentration based on the first isopleth to reach residential areas. This suggested that the annual average ambient air concentration could be about 200 times the IRSL immediately outside the sterilization facility. The results of Phase I justified further examination in Phase II.

**Figure 4:**
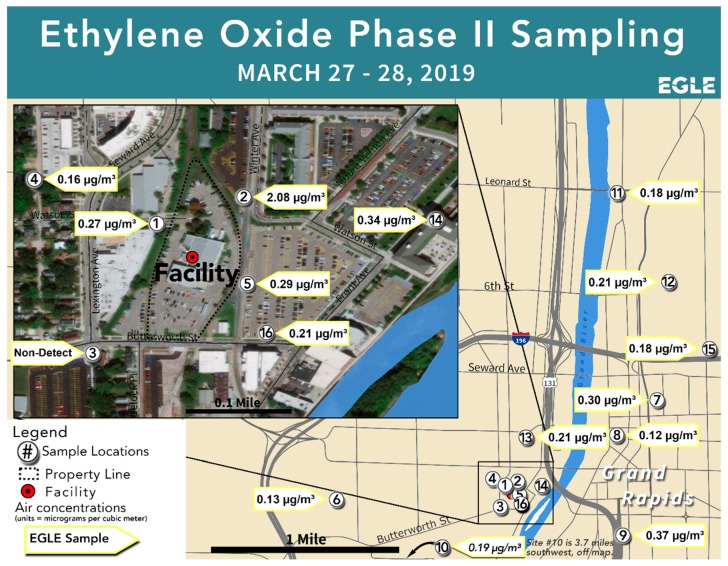
Phase II sampling sites and corresponding ambient air measurements of EtO.

**Figure 5:**
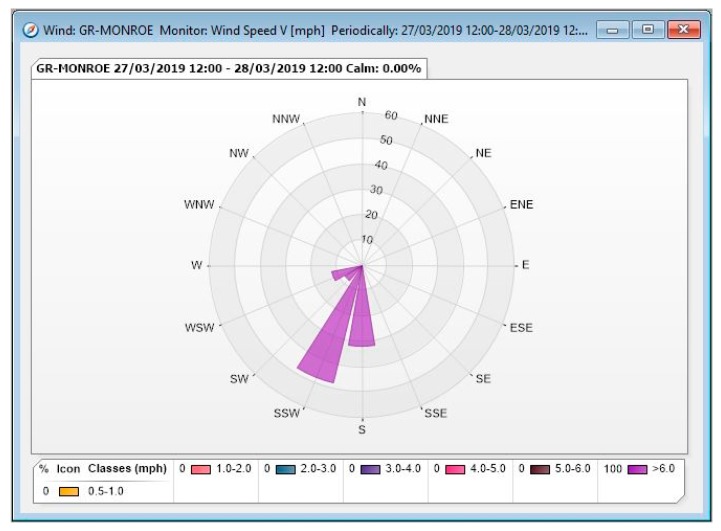
Wind rose for Phase II sampling period based on data from the MDEQ Grand Rapids—Monroe monitoring station.

During Phase II, Summa canisters were set up at 16 locations around the Grand Rapids area, including the campus of Grand Valley State University (GVSU). The locations were mainly downwind, but with a handful of upwind sites. The sampling strategy employed the same quality assurance procedures as in Phase I, including co-located samples at two sites. Once collected, the canisters were prepared for shipment and sent back to the ERG laboratory on March 28, 2019. Table 2 and Figure 4 display the sampling site locations and results, while Figure 5 shows a wind rose for the sampling time period. A second canister at Site #5 yielded the same result as the first co-located canister, while a second co-located canister at Site #8 yielded a non-detect (ND).

The highest 24 h EtO concentration of 2.08 µg/m^3^ occurred in a parking lot immediately across the street from the sterilization facility and directly downwind from the site with the highest concentration during the Phase I sampling. With the exception of one site that had a non-detect (Site #3), all measurement sites showed 24 h EtO concentrations above the IRSL, including sites that were upwind of the facility. This latter finding suggested that there is a background source of EtO in addition to emissions from the sterilization facility itself. 

In Section 3 of this paper, we apply a simple inverse modeling method to further analyze the results of the Phase II campaign. The results of this analysis will answer the questions effectively posed at the end of Section 1, namely:

- Are the emissions reported by the sterilization facility consistent with the canister sampling results?
- How much of the measured ambient exposure to EtO is explained by emissions from the sterilization facility versus background sources?

## 3. Inverse Modeling Analysis

Inverse modeling is a technique that is used to infer the strengths (and possibly the locations) of emission sources from a set of measured impacts around the sources. Olaguer et al. [19] provided examples of how inverse modeling can quantify emissions from natural gas production and transmission facilities based on a 3D Eulerian transport model. Simpler steady-state Gaussian plume models have also been used as the basis for atmospheric inverse modeling [20,21], and this is the approach we have chosen for this study.

Our inverse modeling analysis was simplified by assuming only a single emission source *Q* at a known location (the shipping room vent at the sterilization facility) plus a uniform background concentration *B*, representing the net effect of unknown sources. The assumption of a uniform background concentration over the domain of interest is justified by the atmospheric half-life of EtO, which according to the USEPA, is in the range of 69 days (summer) to 149 days (winter) [22].

Ethylene oxide degrades in the atmosphere mainly by reaction with photochemically produced hydroxyl radicals [23]. Its long lifetime suggests that long-range transport of EtO from distant anthropogenic sources may result in well-mixed urban background concentrations. Ethylene oxide’s atmospheric lifetime is, in fact, similar to that of the globally distributed solvent perchloroethylene, which Olaguer [24] computed to be 105 days on the basis of a 3D global model.

Both *Q* and *B* were inferred from 24 h average EtO measurements obtained during the Phase II canister sampling. The analysis assumes a steady wind field that is the average of all wind measurements at the MDEQ monitoring station in Grand Rapids during the sampling period and ignores the diurnal evolution of the boundary layer height and stability. This latter assumption is justified by the fact that fugitive emissions from the sterilization facility are the dominant source of EtO in the sampling area and are released near the ground rather than at an elevated height. Compared to stack emissions, fugitive emissions should not be as sensitive to assumptions about vertical mixing. In any case, our aim was only to determine the reasonableness of annual emissions reported to MDEQ by the sterilization facility, and not to rigorously compute hourly emissions.

The ground-level EtO concentration field, *C* (µg/m^3^), downwind of the facility, was approximated by the following steady-state formulas:

(1) C(x,y)=B+Q ×D

(2) $$D=\;\frac{exp\left(-\frac{y^{2}}{2\sigma_{y}{}^{2}}\right)}{\pi U\sigma_{y}\sigma_{z}}$$

(3) $$\sigma_{y}=\;\frac{0.16x}{{\left(1+0.0004x\right)}^{0.5}}$$

(4) $$\sigma_{z}=\;\frac{0.14x}{{\left(1+0.0003x\right)}^{0.5}}$$

where:

*x* = distance from facility emission point along wind direction (= 204°)

*y* = perpendicular distance from x-axis

*U* = wind speed (= 4.47 m/s)

*σ_y_* = horizontal dispersion parameter for neutral stability

*σ_z_* = vertical dispersion parameter for neutral stability

Note that our values for the dispersion parameters *σ_y_* and *σ_z_* are adopted from Briggs [25] and are similar to those of Lushi and Stockie [20], except that we assumed urban, rather than rural, conditions.

If *UTM_x_* and *UTM_y_* are the UTM (Universal Transverse Mercator) Zone 16T easting and northing coordinates of a receptor site, and *UTM_x0_* (= 607478.92 m) and *UTM_y0_* (= 4757335.42 m) are the corresponding coordinates for the facility emission point (assumed to be the shipping room vent), then the rotated coordinates *x* and *y* are computed as follows:

(5) $$\left(x_{E},y_{N}\right)=\left(UTM_{x}-\;UTM_{x0}\;,\;UTM_{y}-\;UTM_{y0}\right)$$

(6) $$x=x_{E}\;\cos\;\beta +\;y_{N}\;\sin\beta$$

(7) $$y=-x_{E}\;\sin\;\beta +\;y_{N}\;\cos\beta$$

where β is the wind angle with respect to the x*_E_* axis (=270° − 204°= 66°).

A linear regression technique was used to determine the emission rate and background concentration based on measured values of *C* and computed values of *D*. Only measurement points with a positive value of *x* were used in the linear regression. Site #3 had a non-detect of EtO as well as a negative value of *x*. This site was ignored not only in the linear regression, but also in any further analysis. This resulted in 12 measurement points included in the linear regression. 

The analysis method described above was coded into a Python script and run using the scipy.stats.linregress module. The linear regression yielded estimates for *Q* and *B* of 594 lbs/year and 0.247 µg/m^3^, respectively. The uncertainty in the emissions estimate, based on the standard error of the slope of the linear regression, was 28 lbs/year. The coefficient of determination (R^2^) was 0.9786.

## 4. Discussion

On the basis of indoor air gas chromatograph measurements, the sterilization facility’s estimate of fugitive emissions amounted to 420 lbs of EtO for the entire calendar year of 2018. Our inverse model-inferred value of *Q* is thus within about 41% of the reported annual average emissions. This percent difference is within the normal degree of tolerance for emissions data uncertainty, and so we conclude that the facility-reported estimate is consistent with the Phase II canister sampling results.

The inferred value of *B* can be compared to the average of the EtO concentrations measured at the three sites other than Site #3 (which had a non-detect) that were excluded from the linear regression. Those upwind sites (#6, #10, #16) had an average measured EtO concentration of 0.177 µg/m^3^. Our inferred value of *B* is within 40% of this independent average value. This difference may be roughly interpreted as the uncertainty in the background estimate.

In December 2018, Sterigenics U.S. LLC retained Ramboll to conduct TO-15 testing of background EtO levels in the Chicago metropolitan area [15]. Ramboll collected 35 5 min grab samples and 14 12 h samples. The mean EtO concentration reported for the grab sample locations was 0.22 μg/m^3^, with a standard deviation of 0.11 μg/m^3^. The mean background level estimated from both 5 min and 12 h samples was 0.24 μg/m^3^, with a standard deviation of 0.15 μg/m^3^. Our inferred value of *B* is within 3% of the latter background estimate.

The USEPA likewise conducted 24 h Summa canister sampling at eight sites near the Sterigenics medical sterilization facility in Willowbrook, Illinois [26]. The six-week average EtO background concentration measured by TO-15 analysis after the facility was sealed by the State of Illinois was 0.15 μg/m^3^. In addition, preliminary analysis by the USEPA of EtO concentrations at a subset of the National Air Toxics Trends Stations (NATTS) and Urban Air Toxics Monitoring Program (UATMP) sites for the fourth Quarter of 2018 and first Quarter of 2019 time period yielded an average background concentration of 0.297 μg/m^3^ [26].

Clearly, our inferred estimate of the background concentration of EtO is compatible with estimates based on other similar investigations.

## 5. Conclusions

The Phase II Summa canister sampling conducted by MDEQ revealed a peak 24 h average EtO concentration of 2.08 μg/m^3^ in a residential neighborhood immediately next to the largest medical sterilization facility in Michigan. The facility’s reported annual emissions of EtO roughly agreed with the fugitive emission rate of 594 lbs/year inferred by MDEQ from the Phase II ambient air measurements through inverse modeling. The facility’s contribution to the peak EtO concentration may be roughly assessed by subtracting the inferred background concentration of 0.247 µg/m^3^ from the peak value, so that the sterilization facility is responsible for residential exposure to 1.83 μg/m^3^ of EtO above the background level. This impact corresponds to an additional cancer risk of approximately one in one hundred, if assumed to represent annual mean exposure.

As a result of various inspections and the EtO modeling and monitoring studies conducted by MDEQ, the State of Michigan issued a Violation Notice to the sterilization facility based on Rule 901(a) in Part 55 (Air Pollution Control) of the State of Michigan’s Public Act 451, known as the Natural Resource and Environmental Protection Act (NREPA), which states: “A person shall not cause or permit the emission of an air contaminant in quantities that cause injurious effects to human health or safety.” The facility eventually agreed to permanently shut down its sterilization operations and to pay a significant monetary penalty.

## Figures and Tables

**Table 1:** Phase I sampling results.

Site	Result (µg/m^3^)
1a (co-located)	0.85
1b (co-located)	0.87
2	1.56
3	76.00
4	0.42

**Table 2:** Phase II sampling results. GVSU: Grand Valley State University.

Site	Result (µg/m^3^)
1. Sidewalk, Phase 1 location	0.27
2. GVSU parking lot directly NE of facility (Watson Lot)	2.08
3. 622 Butterworth SW	ND
4. 40 Gold Ave SW	0.16
5. Winter Lot (GVSU) (collocated)	0.29/0.29
6. John Ball Zoo	0.13
7. Crescent Park	0.3
8. Rosa Parks Circle (collocated)	0.12/ND
9. Heartside Park	0.37
10. Clean water plant (upwind site)	0.19
11. GR Monroe St, fixed air monitoring site	0.18
12. Coit Park	0.21
13. Devos parking lot (GVSU)	0.35
14. Seidman Center (GVSU)	0.34
15. Finklestein Center (GVSU)	0.18
16. Bicycle Factory (GVSU)	0.21
